# P-1566. Insights into the Acute Phase of Nipah Virus Infection: Clinical Features, Viral Detection, and Humoral Immune Response

**DOI:** 10.1093/ofid/ofaf695.1746

**Published:** 2026-01-11

**Authors:** Syed Moinuddin Satter, Sharmin Sultana, Shadman Sakib Choudhury, Wasik Rahman Aquib, Dewan Rahman, Mintu Chowdhury, Md Sazzad Hossain, Arifa Nazneen, Kamal Ibne Amin Chowdhury, Anika Farzin, Ayesha Siddika, Fateha Ema, Tonmoy Sarkar, Arifur Rahman Bablu, Muhammad Rashedul Alam, Mohammad Enayet Hossain, Md Taufiqur Rahman Bhuiyan, Trevor Shoemaker, Michael K Lo, Jhon D Klena, Christina Spiropoulou, Mohammed Ziaur Rahman, Sayera Banu, Tahmina Shirin, Mahmuda Yasmin, Firdausi Qadri, Joel M Montgomery, Chowdhury Rafiqul Ahsan

**Affiliations:** icddr,b (International Centre for Diarrhoeal Disease Research, Bangladesh), Dhaka, Dhaka, Bangladesh; Institute of Epidemiology, Disease Control and Research (IEDCR), Dhaka, Dhaka, Bangladesh; icddr,b, Dhaka, Dhaka, Bangladesh; icddr,b (International Centre for Diarrhoeal Disease Research, Bangladesh), Dhaka, Dhaka, Bangladesh; icddr,b, Dhaka, Dhaka, Bangladesh; Institute of Epidemiology, Disease Control & Research (IEDCR), Dhaka, Dhaka, Bangladesh; Institute of Epidemiology, Disease Control & Research (IEDCR), Dhaka, Dhaka, Bangladesh; icddr,b, Dhaka, Dhaka, Bangladesh; icddr,b, Dhaka, Dhaka, Bangladesh; icddr,b (International Centre for Diarrhoeal Disease Research, Bangladesh), Dhaka, Dhaka, Bangladesh; icddr,b (International Centre for Diarrhoeal Disease Research, Bangladesh), Dhaka, Dhaka, Bangladesh; icddr,b, Dhaka, Dhaka, Bangladesh; icddr,b (International Centre for Diarrhoeal Disease Research, Bangladesh), Dhaka, Dhaka, Bangladesh; icddr,b, Dhaka, Dhaka, Bangladesh; icddr,b (International Centre for Diarrhoeal Disease Research, Bangladesh), Dhaka, Dhaka, Bangladesh; icddr,b (International Centre for Diarrhoeal Disease Research, Bangladesh), Dhaka, Dhaka, Bangladesh; icddr,b, Dhaka, Dhaka, Bangladesh; Centers for Disease Control and Prevention (CDC), Atlanta, Georgia; US CDC, Atlanta, Georgia; CDC, Sierra Leone, Greater Accra, Ghana; US CDC, Atlanta, Georgia; icddr,b (International Centre for Diarrhoeal Disease Research, Bangladesh), Dhaka, Dhaka, Bangladesh; icddr,b (International Centre for Diarrhoeal Disease Research, Bangladesh), Dhaka, Dhaka, Bangladesh; Institute of Epidemiology, Disease Control and Research (IEDCR), Dhaka, Dhaka, Bangladesh; University of Dhaka, Dhaka, Dhaka, Bangladesh; International Centre for Diarrhoeal Disease Research, Bangladesh, Dhaka - , Bangladesh; Centers for Disease Control and Prevention (CDC), Atlanta, Georgia; University of Dhaka, Dhaka, Dhaka, Bangladesh

## Abstract

**Background:**

Nipah virus (NiV) infection poses a significant threat to global public health. Understanding its initial acute clinical phase and associated immunological responses may be crucial for assessing prognosis as well as developing effective treatment strategies.

**Table 1:**
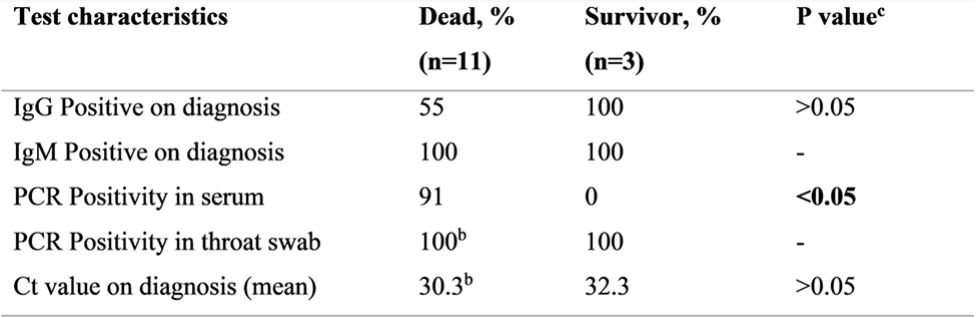
Laboratory test results summary among confirmed Nipah fatal and survivors cases (N=14)* *: 14 out of 15 cases were assessed for serology, as 1 patient died before serum sample collection b: n=15 for throat swabs (12 dead vs 3 survivors), as the throat swab was acquired from the patient who died before serum collection c: p values were obtained using Fisher’s Exact test for categorical variables, and t-test for continuous variables

**Figure 1:**
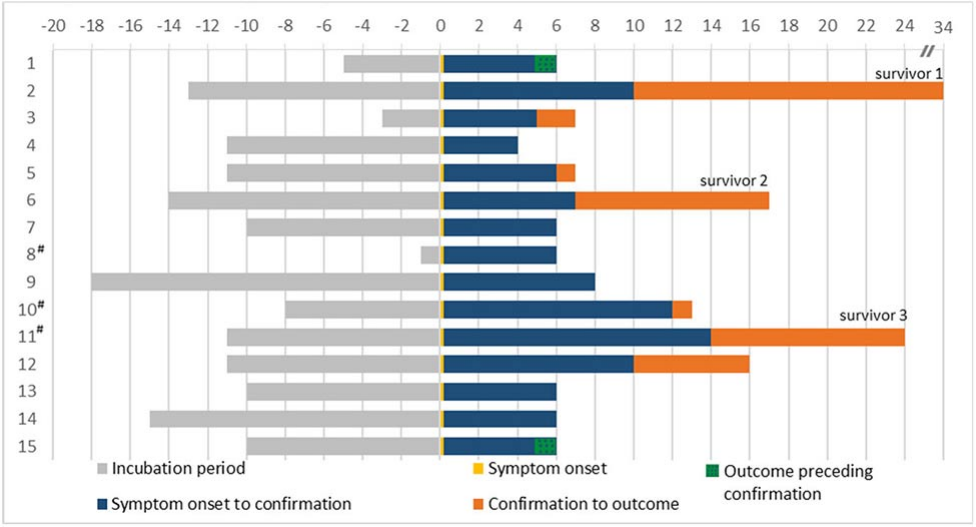
Timelines of confirmed cases from exposure to death/discharge in days since symptom onset (N=15) # : secondary cases

**Methods:**

During the 2023 and 2024 NiV outbreaks in Bangladesh, clinical and laboratory data from 15 confirmed cases were collected and used for this study. Throat swabs and serum samples were tested for NiV genomic material by real-time reverse transcriptase polymerase chain reaction (rT-PCR); patient humoral immune response was detected by enzyme-linked immunosorbent assay (ELISA) for immunoglobulin M and G.

**Figure 2:**
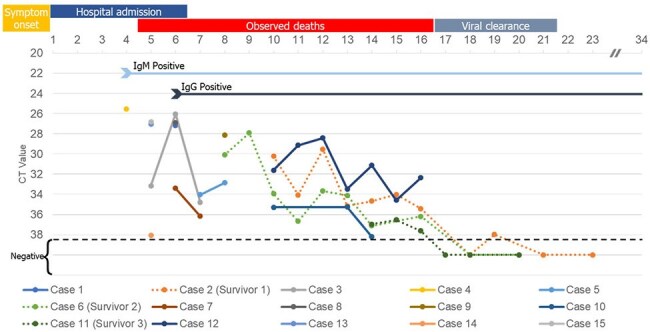
Clinical and laboratory events observed across days since illness onset IgM positive from day 4, and IgG positive from day 6. Hospital admissions occurred within the 1st-6th day post onset of symptoms. All deaths were observed during the 5th-16th day of POS. Viral clearance from throat swabs was noted in 3 survivors during the 17th-21st day POS. The overall trend shows a gradual increase in CT values.

**Figure 3:**
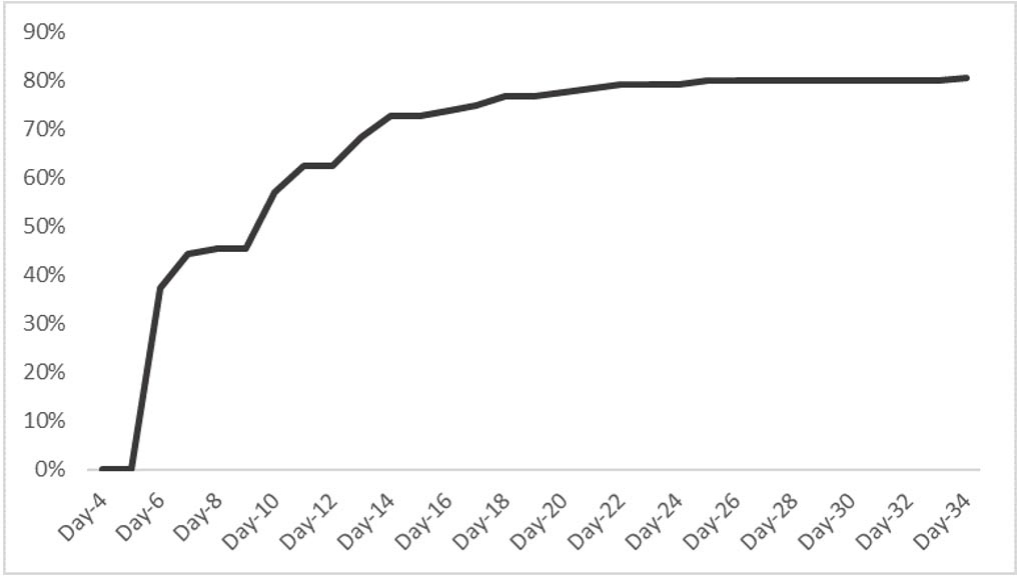
Cumulative test positivity of IgG among Nipah confirmed cases (N=14)

**Results:**

Cases were evenly distributed between genders, with a median age of 18 (0-65) years. The case fatality rate (CFR) for the 15 cases was 80% (12/15), with a median survival duration of 6 (3-16) days since illness onset among the deceased. Twelve (80%) of the cases had evidence of primary infection, with all having had a history of raw date palm sap (DPS) consumption within 28 days preceding symptom onset. The median incubation period among primary cases was 11 days (range: 3-19 days), 3 days longer than that of secondary infection cases. Survivors exhibited a longer median incubation period of 13 (11-14) days compared to fatal cases for whom it was 10 (1-19) days. Serum samples from survivors tested by PCR were negative, indicating no evidence of viremia on diagnosis, whereas 92% (10/11) of the fatal cases that could be tested for serology tested positive with their primary diagnostic sample. Anti-NiV IgM and IgG were detectable as early as the fourth and sixth day post-symptom onset, respectively, and as late as the 34^th^ day. All survivors tested IgG positive on diagnosis compared to only half of the fatal cases.

**Conclusion:**

The study provides critical insights into the clinical indices, immune response, and viral detection during NiV infection. This could be pivotal in predicting clinical outcomes and guiding treatment strategies for NiV infection.

**Disclosures:**

All Authors: No reported disclosures

